# Association between Dietary Pattern, Lifestyle, Anthropometric Status, and Anemia-Related Biomarkers among Adults: A Population-Based Study from 2001 to 2015

**DOI:** 10.3390/ijerph18073438

**Published:** 2021-03-26

**Authors:** Rathi Paramastri, Chien-Yeh Hsu, Hsiu-An Lee, Li-Yin Lin, Adi Lukas Kurniawan, Jane C.-J. Chao

**Affiliations:** 1 School of Nutrition and Health Sciences, College of Nutrition, Taipei Medical University, 250 Wu-Hsing Street, Taipei 11031, Taiwan; rara.paramastri@gmail.com; 2 Department of Information Management, National Taipei University of Nursing and Health Sciences, 365 Ming-Te Road, Peitou District, Taipei 11219, Taiwan; cyhsu@ntunhs.edu.tw; 3 Master Program in Global Health and Development, College of Public Health, Taipei Medical University, 250 Wu-Hsing Street, Taipei 11031, Taiwan; 4 Department on Computer Science and Information Engineering, Tamkang University, 151 Yingzhuan Road, Tamsui District, New Taipei 25137, Taiwan; billy72325@gmail.com; 5 National Health Research Institutes, 35 Keyan Road, Zhunan Town, Miaoli County 35053, Taiwan; 6 School of Public Health, College of Public Health, Taipei Medical University, 250 Wu-Hsing Street, Taipei 11031, Taiwan; jlin11025@gmail.com; 7 Research Center for Healthcare Industry Innovation, National Taipei University of Nursing and Health Sciences, 365 Ming-Te Road, Peitou District, Taipei 11219, Taiwan; 8lukas@ntunhs.edu.tw; 8 Nutrition Research Center, Taipei Medical University Hospital, 252 Wu-Hsing Street, Taipei 11031, Taiwan

**Keywords:** dietary pattern, reduced rank regression, anemia, anthropometric data, lifestyle, adults

## Abstract

Inadequate dietary intake, poor nutritional status, heavy smoking, and alcohol consumption are associated with the risk of anemia. The objective of this study was to investigate the associations between dietary patterns, lifestyle, nutritional status, and anemia-related biomarkers among adults using a multivariable regression model. Taiwanese adults aged 20–45 years (*n* = 118,924, 43,055 men and 75,869 women) were obtained from the Mei Jau Health Management Institution database, between 2001 and 2015, for data analysis. The anemia–inflammation-related dietary pattern was derived by reduced rank regression analysis. Dietary patterns with high intakes of eggs, meat, organ meats, rice or flour products, fried foods, sugary beverages, and processed foods significantly increased the risk of anemia, and was associated with decreased hemoglobin, hematocrit, and red blood cells, but increased white blood cells and C-reactive protein levels. Moreover, current alcohol drinkers, as well as people who were underweight, overweight, obese, and central obese, were more likely to increase their risk of anemia by 46%, 20%, 23%, 34%, and 28%, respectively. Interestingly, participants who are current or past smokers were inversely associated with risk of anemia. In conclusion, adherence to the anemia–inflammation dietary pattern was associated with an increased risk of anemia in Taiwanese adults. Furthermore, abnormal weight status and alcohol drinking were correlated with an increased risk of anemia.

## 1. Introduction

Anemia is defined as a condition where hemoglobin (Hb) concentration is less than the requirement, and has been known to be a global health problem with significant adverse health consequences [1]. The clinical definition of anemia by the World Health Organization (WHO) is Hb ≤ 7.45 mmol/L (12 g/dL) in women and Hb ≤ 8.07 mmol/L (13 g/dL) in men [2]. Although the most reliable indicator of anemia at the population level is hemoglobin concentration, measurement of this parameter alone cannot determine the cause of anemia [3]. Anemia has affected more than 2 billion people globally, which is approximately over a quarter of the world’s population [4]. Cohort survey data from National Health and Nutrition Examination Survey, from 2003 to 2012, in the United States showed that the overall prevalence of anemia was 5.6%, and the rate of moderate/severe anemia was 1.5%, where pregnant women were excluded [5]. A repeated cross-sectional survey in rural China among adults aged 18–64 years demonstrated that the prevalence of anemia was 51.5% and 53.7% in 2006 and 2008, respectively, and that women had a significantly higher prevalence of anemia than men in any age group in both 2006 and 2008 [1]. A cohort study from January 2012 to January 2013 in rural south-west Uganda showed that the prevalence of anemia was 16.8% and 17.6% in men and women aged 50–64 years, respectively [6]. A nutrition and health survey in Taiwan from 2005 to 2008 revealed that the prevalence of anemia was lower than 10% in men and close to 20% in women aged 19–44 years [7].

The previous studies showed that inadequate dietary intake, poor nutritional status, heavy smoking or alcohol consumption, and sleep disturbance or duration, were correlated with iron metabolism or the risk of anemia [6,8,9,10,11,12]. Less fruit intake [6] and those who were underweight [8] were associated with an increased risk of having anemia. However, people who were actively smoking increased Hb by 0.16–0.37 mmol/L (0.26–0.59 g/dL), depending on the number of cigarettes smoked [9]. A cross-sectional study in Korea between February 2011 and November 2012 found that heavy drinkers aged 49–79 years with >30 g/d alcohol consumption had increased odds of iron overload by 61% (OR = 1.61, 95% CI 1.11, 2.36) compared to non-drinkers [10]. An epidemiological study in the UK reported that people aged ≥50 years with intermediate sleep disturbance were more likely to have anemia (OR = 1.59–1.73, 95% CI 1.02–1.13, 2.46–2.65) compared to those with low sleep disturbance [11]. Additionally, a prospective cohort study in the Kailuan community from June 2006 to October 2007 among Chinese aged ≥18 years revealed that people with sleep durations of ≤5 h, 6 h, and ≥9 h significantly increased hazard ratios of anemia by 23% (HR = 1.23, 95% CI 1.04, 1.45), 26% (HR = 1.26, 95% CI 1.11, 1.44), and 42% (HR = 1.42, 95% CI 1.08, 1.86), respectively, compared to those with a sleep duration of 7 h [12].

Current knowledge about the linkage between dietary factors and anemia has mainly focused on individual nutrients. The dietary pattern is currently considered as a new approach to be applied in nutritional epidemiology for the assessment of the association between dietary factors and disease risk [13]. Several previous studies examined the association between overall dietary patterns and anemia by considering how foods and nutrients are consumed in combinations [14,15]. However, the study for the relationship between dietary patterns and anemia in adults was still limited in Taiwan. Therefore, we aimed to explore the associations of dietary patterns, lifestyle variables, and anthropometric status with anemia in Taiwanese adults.

## 2. Materials and Methods

### 2.1. Data Source and Study Participants

**Figure 1:**
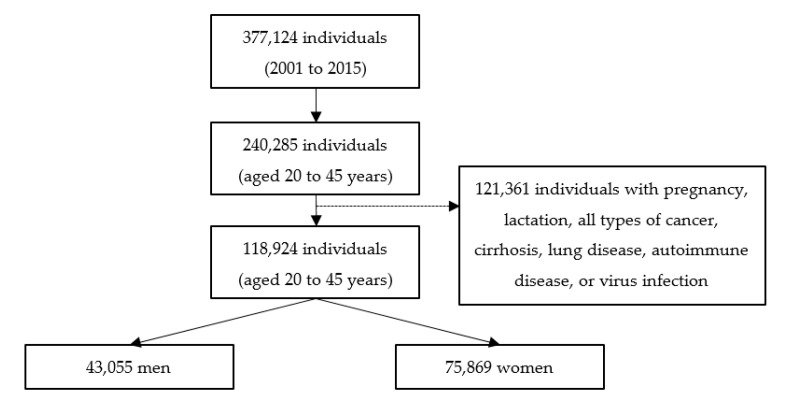
Flowchart of study participants.

This study used data between 2001 and 2015 from the Mei Jau (MJ) Health Management Institution, which has four health screening centers located in Taipei, Taoyuan, Taichung, and Kaohsiung, in Taiwan. Subjects who visited the health screening center for health examinations were requested to fill in the self-reported questionnaire for information on sociodemographic data, lifestyles, medical history, and dietary habits, and have anthropometric measurements and blood tests taken, after at least 8-h of fasting, by a trained nurse or medical laboratory technician. All subjects signed a consent form and allowed the MJ Health Management Institution use of their information for research purpose, without personal identification. A total of 118,924 subjects (43,055 men and 75,869 women) aged 20–45 years were included in this study for analysis. We excluded those who were pregnant or breast-feeding, and those who had any type of cancer, cirrhosis, lung disease, autoimmune disease, or viral infection (Figure 1). The Joint Institutional Review Board of Taipei Medical University (TMU-JIRB N201907025) approved this study.

### 2.2. Anthropometric and Biochemical Data

The anthropometric data, including body weight, height, and waist and hip circumferences, were retrieved from the database. Body weight and height of the subjects were measured by using an auto-anthropometer (KN-5000A, Nakamura, Tokyo, Japan). Body mass index (BMI) was calculated by kg/m2. Weight status is defined by using BMI criteria in Taiwan: underweight (BMI < 18.5 kg/m^2^), normal weight (18.5 kg/m^2^ ≤ BMI < 24 kg/m^2^), overweight (24 kg/m^2^ ≤ BMI < 27 kg/m^2^), or obese (BMI ≥ 27 kg/m^2^) [16]. Waist circumference (WC) was used to define central obesity, with WC ≥ 90 cm for men and ≥80 cm for women [17]. Biochemical data were analyzed by the central laboratory of the MJ Health Management Institution. Subjects completed blood tests (Abbott Cell-Dyn 3700 hematology analyzer, Abbott Park, IL, USA) when they visited the health screening center for health examinations. Biochemical data, including anemia-related biomarkers such as hemoglobin (Hb), hematocrit (Hct), and red blood cells (RBC), as well as inflammation biomarkers such as white blood cells (WBC) and C-reactive protein (CRP), were retrieved from the database. The level of CRP was measured using an auto-analyzer (Toshiba C8000, Tokyo, Japan). The definition of anemia was based on WHO criteria: Hb < 8.07 mmol/L (13 g/dL) and <7.45 mmol/L (12 g/dL) for men and women, respectively [2], and hematocrit level <33% [18].

### 2.3. Dietary Assessment and Other Covariates

The MJ Health Management Institution had developed a standardized and validated semi-quantitative food frequency questionnaire (FFQ) with 22 food groups, and which was reported in previous studies [19,20]. The initial questionnaire comprised 85 closed-ended questions on individual food items, and was further classified into 22 non-overlapping food groups on the basis of presumed health effects and similarity, as described in previous studies [21,22,23]. Frequency of a fixed serving consumed was assessed per day, or in a week in the past, one month prior to data collection. There were 5 response options for each food group, as described previously [21]. Dietary data were collected for further analysis to derive dietary patterns using a reduced rank regression model. A self-reported questionnaire, including demographic data, lifestyles, and medical history, was used to collect personal information. Demographic and lifestyle characteristics included age, gender, smoking (non-smoker, past smoker, or current smoker), drinking alcohol (no or yes with ≥1 time a week), chewing betel nut (no or yes with ≥1 time a week), sleep duration (<6 h, 6–8 h, or >8 h), and physical activity (≤2 h a week or >2 h a week). Demographic and lifestyle variables were used for association analysis with anemia or anemia–inflammation-related biomarkers, and for the adjusted variables in the models of regression analysis. Medical history included the use of drugs for hypertension, diabetes, cardiovascular disease, or hyperlipidemia.

### 2.4. Statistical Analysis

**Figure 2:**
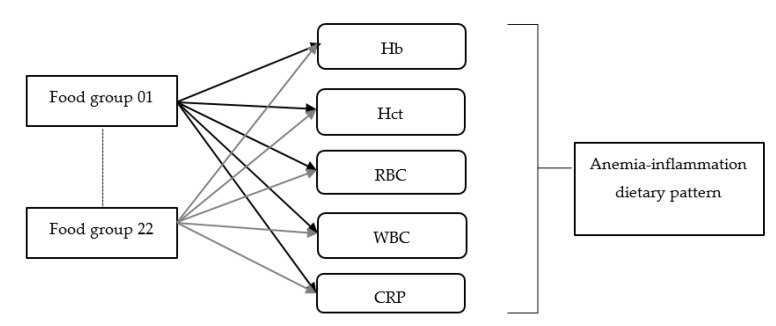
The dietary pattern derived from the reduced rank regression model. Hb: hemoglobin, Hct: hematocrit, RBC: red blood cells, WBC: white blood cells, CRP: C-reactive protein.

Statistical analysis was performed using SAS version 9.4 (SAS Institute, Chicago, IL, USA). A Kolmogorov–Smirnov test was performed to determine the distribution of the data. A Mann–Whitney U test was used to compare the differences between two groups. Furthermore, differences among multiple groups were assessed by one-way analysis of variance (ANOVA), followed by a Kruskal–Wallis test. A chi-square test was used to analyze the categorical data. The multivariable logistic regression was performed to identify the risk factors associated with anemia. The multivariable linear regression was conducted to determine the association of dietary patterns, lifestyle, and anthropometric data with anemia-related biomarkers. Dietary pattern was derived by reduced rank regression (RRR) with the PROC PLS function using SAS 9.4, and 22 food groups were used as predictors. We included Hb, Hct, RBC, WBC, and CRP for the response variables (Figure 2). According to previous investigations, we retained all food groups which had the absolute value of factor loading ≥0.20 to derive the dietary patterns associated with anemia [24,25]. Then, we calculated dietary factor scores for each food group by summing food frequency intake weighed by their factor loadings. Finally, we only retained the first factor which explained the greatest variation in the response variables. The derived dietary pattern was divided into tertiles according to dietary factor scores. The *p*-value < 0.05 was considered statistically significant.

## 3. Results

### 3.1. Characteristics of the Study Participants

The characteristics of the subjects are presented in Table 1. A total of 10.8% (*n* = 12,901) of the subjects had anemia among all subjects (*n* = 118,924) aged 20–45 years. The majority of anemic subjects were female (96.0%). Women had a higher anemia prevalence than men (16.3% vs. 1.2%). The characteristics of the subjects were more likely to be not smoking, not drinking, not chewing betel nut, having a high proportion of short sleep duration (<6 h), sedentary activity (≤2 h/week), and normal BMI. The prevalence of hypertension and diabetes was 4.6% and 1.3%, respectively. Anemic subjects were less likely to be current smokers, drinkers, betel nut chewers, suffer from short sleep duration (<6 h), engage in active physical activity (>2 h/week), have hypertension, be overweight, obese, or have central obesity, and they had worse anemia biomarkers and a higher WBC count compared to those without anemia.

### 3.2. Anemia-Inflammation Dietary Pattern

**Figure 3:**
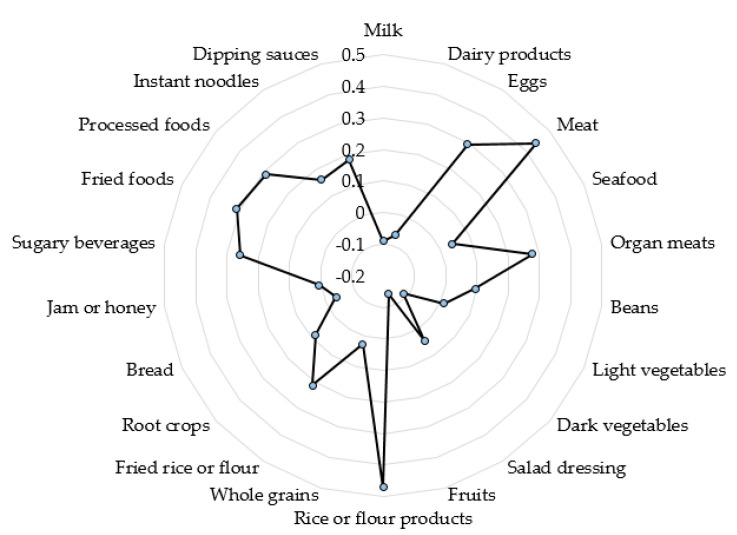
Spider web diagram of anemia–inflammation-related dietary pattern with factor loadings.

The RRR model identified one specific dietary pattern related to anemia. The anemia–inflammation-related dietary pattern with absolute factor loadings of ≥0.20 was characterized by high intakes of eggs, meat, organ meats, rice or flour products, fried rice or flour, sugary beverages, fried foods, and processed foods (Figure 3). The first factor derived by RRR explained 6.2% of Hb, 6.1% of Hct, 5.2% of RBC, 1.2% WBC, 0.1% CRP, and 8.6% of the total variation in 5 response variables.

### 3.3. Association of Lifestyle and Anthropometric Data with Anemia

Table 2 demonstrated the association of lifestyle and anthropometric data, with anemia analyzed by the multivariable logistic regression. In the unadjusted model, smoking was negatively associated with anemia (OR = 0.29, 95% CI 0.27, 0.32 for past smokers; OR = 0.43, 95% CI 0.38, 0.49 for current smokers). However, drinking alcohol (OR = 2.55, 95% CI 2.31, 2.81) and inactive physical activity (OR = 1.38, 95% CI 1.31, 1.46) were positively associated with anemia. After adjusting for age, gender, hypertension, and diabetes, smoking and drinking alcohol remained significantly associated with anemia. Subjects who were past or current smokers decreased the risk of anemia by 32% (OR = 0.68, 95% CI 0.63, 0.74) or 26% (OR = 0.74, 95% CI 0.64, 0.86), respectively. By contrast, active alcohol drinkers increased the risk of anemia by 46% compared to those who were non-drinkers (OR = 1.46, 95% CI 1.32, 1.61).

The multivariable logistic regression showed that abnormal BMI was positively associated with the risk of anemia in both crude and adjusted models (Table 2). After adjusting for the full model (model 2), subjects who were underweight (OR = 1.20, 95% CI 1.10, 1.42), overweight (OR = 1.23, 95% CI 1.10, 1.38), or obese (OR = 1.34, 95% CI 1.22, 1.48) significantly increased the risk of anemia by 20%, 23%, or 34%, respectively, compared to those with normal BMI. Additionally, subjects with central obesity increased the risk of anemia by 28% (OR = 1.28, 95% CI 1.18, 1.39) in model 2.

### 3.4. Association between Lifestyle, Anthropometric Data, and Anemia-Related Biomarkers

Table 3 illustrated the associations between lifestyle, anthropometric data, and anemia or inflammation-related biomarkers. Subjects who had a history of smoking increased Hb level (β = 0.18, 95% CI 0.17, 0.19, *p* < 0.05) and WBC count (β = 0.13, 95% CI 0.07, 0.19, *p* < 0.001) compared to those who were non-smokers. Similarly, subjects who were current smokers had higher Hb, Hct, WBC, and CRP levels (*p* < 0.001). However, alcohol drinkers showed decreased Hb levels (β = −0.08, 95% CI −0.09, −0.06, *p* < 0.001) and RBC count (β = −0.05, 95% CI −0.05, −0.04, *p* < 0.001) compared to non-drinkers. Short sleep duration (<6 h) decreased Hb level (β = −0.02, 95% CI −0.03, −0.01, *p* < 0.05) and increased WBC count (β = 0.28, 95% CI 0.03, 0.53, *p* < 0.05) compared to normal sleep duration (6–8 h), while long sleep duration (>8 h) did not show any significant associations with anemia or inflammation-related biomarkers. Subjects who were physically inactive (>2 h/week) had lower Hb, Hct, and RBC levels (all *p* < 0.001), but higher WBC and CRP levels (all *p* < 0.05) compared to those with active physical activity (>2 h/week). Being underweight was negatively associated with RBC and WBC counts (both *p* < 0.001). Being overweight, obese, or having central obesity was negatively correlated with anemia-related biomarkers, but positively associated with inflammation biomarkers (all *p* < 0.001).

### 3.5. Association between Dietary Pattern, Anemia, and Anemia-Related Biomarkers

The associations between dietary patterns across tertiles and the risk of anemia, or anemia-related biomarkers, are shown in Table 4. Subjects in the highest tertile (T3) of the dietary pattern increased the risk of anemia by 87% (OR = 1.87, 95% CI 1.78, 1.95, *p* < 0.001) in the crude model (model 1) compared to those in the lowest tertile (T1) of the dietary pattern. After adjusting for age, gender, lifestyle, history of chronic diseases, and weight status (model 3), subjects in the highest tertile of the dietary pattern increased the risk of anemia by 59% (OR = 1.59, 95% CI 1.51, 1.67, *p* < 0.001). In addition, subjects in the highest tertile of the dietary pattern had worse anemia biomarkers and increased WBC and CRP levels (all *p* < 0.001), in all the models, compared to those in the lowest tertile of the dietary pattern.

## 4. Discussion

### 4.1. Lifestyle and Anemia

Our study found that past or current smokers decreased the risk of anemia, and current smokers were more likely to increase Hb and Hct levels. Similarly, the previous studies showed that smoking was negatively correlated with the risk of anemia [26,27,28]. The multiple logistic regression analysis from the health check-up database of St. Luke’s international hospital in Tokyo, between April 2016 and March 2017, revealed that Japanese women (35–49 years) who were past or current smokers decreased the risk of anemia by 33% (OR = 0.67, 95% CI 0.56, 0.81, *p* < 0.001) or 25% (OR = 0.75, 95% CI 0.56, 0.99, *p* = 0.045), respectively, compared to those who were non-smokers, after adjusting for the covariates [26]. However, the association between past or current smoking and anemia was not statistically significant in Japanese women aged 20–34 years, in both unadjusted and adjusted models [26]. A previous study in India found that male smokers aged 30–60 years had significantly higher Hb, Hct, and RBC levels compared to male non-smokers [27]. Additionally, an increase in the severity of smoking in adult male smokers showed significantly increased Hb, RBC, mean corpuscular volume, and mean corpuscular Hb concentration [28]. The pack-years of smoking were also positively correlated with Hb (*r* = 0.418, *p* < 0.001) and RBC levels (*r* = 0.215, *p* = 0.03) in adult male smokers [28]. Although this finding should not be interpreted as an alternative approach to decrease the risk of anemia, the cut-off value for Hb should be adjusted to diagnose anemia in smokers; this is because the Hb distribution curve shows an upward shift with smoking [29,30]. The WHO released a guideline for the definition of anemia by Hb cut-off values, with adjustment for smoking and altitude, in 2001 [2]. Increased Hb in smokers was associated with elevated carboxyhemoglobin (HbCO), a stable complex of Hb and carbon monoxide (CO), because of exposure to excess CO caused by smoking [29]. The form of HbCO decreases oxygen delivery, and smokers had elevated Hb as a compensatory mechanism to increase erythropoiesis rate and maintain oxygen transportation [27,30]. This might explain why adaption to excess CO during smoking was reflected by the rise in Hb and RBC mass [31]. Furthermore, increased Hb and RBC could be responsible for an elevation of Hct levels in smokers [27].

Our findings showed that alcohol drinking increased the risk of anemia, and was correlated with reduced Hb and RBC levels. Consistent with our results, the previous studies demonstrated a positive correlation between alcohol drinking and anemia [32,33]. A case study in the UK observed that a 56-year-old type 2 diabetic male with chronic alcohol abuse for more than 10 years experienced severe anemia [32]. A previous study in India found that subjects aged ≥ 18 years with moderate (alcohol < 11 drinks/day) to severe intakes (alcohol ≥ 11 drinks/day) of alcohol had lower Hb, RBC, and mean corpuscular Hb concentrations (*p* < 0.001) compared to those who did not drink alcohol [33]. However, Japanese women (20–49 years) who were habitual drinkers decreased the risk of anemia by 27%−33% (20–34-year women: OR = 0.67, 95% CI 0.48, 0.92, *p* = 0.015, 35–49-year women: OR = 0.73, 95% CI 0.64, 0.83, *p* < 0.001) compared to those who were non-drinkers, after adjusting for the covariates [28]. The direct causality of this negative correlation between alcohol drinking and anemia, in the previous study, is still unclear. Heavy alcohol consumption can decrease the number of RBC precursors in bone marrow, and generally affect the formation of functional RBC by interfering with the maturation of RBC [34,35]. The presence of defective RBC by exposure to excess alcohol can contribute to anemia in alcoholic individuals [35].

### 4.2. Anthropometric Data and Anemia

In our study, all abnormal weight statuses, including underweight, overweight, obesity, and central obesity, were linearly associated with an increased risk of anemia. The present study suggested that being underweight was only associated with decreased levels of RBC and WBC in the multivariable-adjusted model. The findings are in concordance with the previous studies [8,36]. A cross-sectional study in India showed that undergraduate medical students who were underweight (BMI < 18 kg/m^2^) increased the risk of anemia by 7.07-fold compared to those with normal BMI (18.0–22.9 kg/m^2^), after adjusting for the covariates (OR = 7.07, 95% CI 1.34, 37.26, *p* = 0.021) [8]. Additionally, a multivariate logistic regression revealed that severely underweight women (BMI ≤ 17.5 kg/m^2^) aged 20–39 years in Aichi, Japan, were more likely to increase the risk of low lymphocytes (<1500/μL) compared to those with normal BMI (18.5–24.9 kg/m^2^) (OR = 1.95, 95% CI 1.07, 3.56, *p* = 0.03) [36]. Young women aged 16–35 years who were underweight (16–20 years: BMI < 5th percentile, 21–35 years: BMI < 18.5 kg/m^2^) in a rural area of East Java, Indonesia, did not significantly increase the risk of anemia (OR = 2.34, 95% CI 0.97, 5.67, *p* = 0.060), but were more likely to have iron depletion (OR = 5.88, 95% CI 2.02, 17.09, *p* = 0.001) and iron-deficient erythropoiesis (OR = 4.52, 95% CI 1.77, 11.54, *p* = 0.002) compared to those who were not underweight [37]. Our study observed that being overweight, obese, and having central obesity were correlated with increased odds of anemia, as well as decreased Hb, Hct, and RBC levels but increased WBC and CRP levels. However, the previous study showed that overweight Japanese women (BMI: 25.0–29.9 kg/m^2^) aged 35–49 years were more likely to have a reduced risk of anemia (OR = 0.74, 95% CI 0.59, 0.92, *p* = 0.007) compared to those with normal BMI (18.5–24.9 kg/m^2^); there was no significant association between obesity and anemia [26].

The associations between being overweight, obese, or having central obesity and anemia could be explained by the rise of inflammatory activity in adipose tissue in relation to impaired iron homeostasis in overweight/obese individuals [38]. Increased inflammatory cytokines in overweight or obese individuals could elevate hepcidin, which binds to cellular ferroportin, an iron exporter, and induces the degradation of ferroportin [39,40]. The degradation of ferroportin leads to the inhibition of iron transport and a decrease of iron in circulation due to iron retention within the macrophage, which results in anemia of inflammation [40]. In concordance with our results, a cross-sectional study from the data of a 2003–2004 National Health and Nutrition Examination Survey in the US found that heavier female adolescents (BMI ≥ 85th percentile) aged 12–17 years had a higher prevalence of iron deficiency (30.8% vs. 14.0%, *p* = 0.003) compared to those with normal weight status (5th percentile ≤ BMI < 85th percentile) [41]. However, dietary iron intake was not significantly different between heavier and normal weight female adolescents (median: 12.10 vs. 13.40 mg/d, *p* = 0.296) [41]. Therefore, even with adequate iron intake, overweight or obese individuals with excess body fat appeared to have a higher risk of developing iron deficiency anemia [41].

### 4.3. Dietary Pattern and Anemia

Our study identified the anemia–inflammation-related dietary pattern is characterized by higher consumption of eggs, meat, organ meats, rice or flour products, fried rice or flour, sugary beverages, fried foods, and processed foods, but lower intakes of vegetables and fruits. In the multivariable logistic regression model, this dietary pattern was significantly associated with an increased risk of anemia and worse anemia-related biomarkers. The characteristics of the anemia–inflammation-related dietary pattern were similar to those of the Western dietary pattern, including high intakes of meat, processed meat, refined grains, fried foods, and sugary foods [42]. A cross-sectional study showed that the Western pattern was correlated with increased prevalence ratios (PR) of anemia (girls: PR = 1.24, 95% CI 1.06, 1.45) and being overweight/obese (boys and girls: PR = 1.15, 95% CI 1.08, 1.21) in Mexican adolescents aged 12–19 years [43]. Additionally, a previous study performed with Thai girls aged 13–15 years reported that 85.5% students consumed dietary iron at <67% of the Recommended Daily Allowance, and most students ate fewer green vegetables and fruits with their meal and drank more sweetened juice [44]. The Western dietary pattern has been shown to have inflammatory effects, according to the previous study by Kurniawan et al. [14]. The inflammatory dietary pattern, with high intakes of eggs, meat, preserved/processed foods, and sugary beverages, but low intakes of grains, vegetables, and fruits, was associated with an increased risk of anemia by 28–47% in middle-aged and older Taiwanese adults with impaired kidney function [14]. High adherence to the Western dietary pattern, and the status of being overweight or obese, could be associated with low-grade inflammation, which leads to a higher incidence of anemia. In agreement with our study, the anemia–inflammation-related dietary pattern and excess adiposity were positively correlated with an increased risk of anemia.

Our study observed a positive correlation between the anemia–inflammation-related dietary pattern and inflammatory biomarkers, such as WBC and CRP. Anemic subjects had a higher WBC count than non-anemic subjects, but CRP levels were not significantly different between anemic and non-anemic subjects. Similarly, the RRR-derived, or partial least squares regression-derived, Western dietary pattern, commonly characterized by high intakes of fried foods, processed foods, and sugary beverages, but low intakes of vegetables, fruits, and soup, was positively associated with CRP levels (*r* = 0.39 or *r* = 0.32) in non-menopausal women aged 42–52 years [45]. Moreover, chronic obstructive pulmonary disease (COPD) patients with anemia had significantly higher CRP levels compared with the control subjects or those without anemia (both *p* < 0.001) [46]. The association between Hb and CRP was also significantly negative in all COPD patients (*r* = −0.28, *p* < 0.01) [46]. Patients with iron deficiency anemia had higher CRP levels compared to those with pernicious anemia [46]. These findings indicated that CRP as an inflammatory marker was inversely correlated with Hb, and might be a predictor for the risk of anemia.

In addition, the “sweet tooth” dietary pattern, characterized by a high consumption of sweetened beverages, milk, and cake, was more likely to increase the prevalence of anemia [47]. The highest quartile (Q4) of the “sweet tooth” dietary pattern increased the odds of anemia in men (OR = 2.34, 95% CI 1.47, 3.73, *p* < 0.001) and women (OR = 2.02, 95% CI 1.31, 3.13, *p* = 0.002) compared to the lowest quartile (Q1) of the dietary pattern [45]. The possible mechanism for the association of dietary pattern and anemia, particularly iron deficiency anemia, could be correlated with the effects of dietary components on the absorption or bioavailability of iron [47]. The bioavailability of iron depends on several factors, including the form of iron (heme vs. non-heme iron), the amount of iron in the diet, the presence of iron enhancers or inhibitors in the diet, and iron status in the individual [48]. Eggs inhibits non-heme iron absorption [49], and rice high in phytate (1.85–9.63 g/kg rice) [50] inhibits iron absorption by chelating with iron [51]. However, meat has some beneficial roles in iron homeostasis, not only due to its heme content but, also, as an enhancer for non-heme iron absorption [47]. Although the dietary pattern derived in our study was high in meat and organ meats, the presence of certain other food groups, such as eggs and rice, in this dietary pattern may interfere with iron absorption or bioavailability.

### 4.4. Strengths and Limitations

The current study has several strengths. To the best of our knowledge, this is the first study to investigate the associations between a dietary pattern determined by an RRR method and anemia-related inflammation in Taiwanese adults. A dietary pattern is a new approach for evaluating eating patterns in the general population [13]. The use of an RRR to derive the dietary pattern provides a better explanation for diet–disease associations [24]. Additionally, our study highlighted the correlation of several variables, including dietary pattern, lifestyle, and anthropometric data, with anemia. Furthermore, we had a large study population that may generalize the results at a greater scale.

In the present study, a number of limitations should be considered. First, it was limited by its cross-sectional study design, which cannot determine a causal relationship. Second, there was a possibility that the use of self-reported questionnaires could lead to misreporting food consumption. Third, the RRR method, even as a complimentary approach with a better reflection of diet and its relationship to diseases, requires response variables (such as biomarkers) in the regression model, which may not completely reflect the current state of knowledge. Indeed, there are still possibly undiscovered parameters as response variables, which may be associated with anemia. Fourth, we only defined anemia generally, according to WHO criteria, in this study, but cannot identify the type of anemia from the original database. Finally, our study had adjusted the results with classic confounders, but there are still some potential confounders, such as altitude, economic status, and mineral and vitamin intake, which can be considered in the future study.

## 5. Conclusions

The RRR-derived dietary pattern, characterized by high intakes of eggs, meat, organ meats, rice or flour products, fried foods, sugary beverages, and processed foods, was associated with an increased risk of anemia. Moreover, the highest tertile of the anemia–inflammation-related dietary pattern was more likely to decrease Hb, Hct, and RBC levels but increase WBC and CRP levels compared to the lowest tertile of the dietary pattern. Current smoking was correlated with increased anemia and inflammatory biomarkers. However, alcohol drinking was associated with decreased Hb and RBC. Short sleep duration (<6 h) was correlated with decreased Hb but increased WBC. Inactive physical activity (≤2 h/week) was associated with reduced anemia biomarkers but elevated inflammatory biomarkers. Our study also suggested that abnormal weight status was correlated with an increased risk of anemia and decreased anemia biomarkers, but increased inflammatory biomarkers.

## Figures and Tables

**Table 1:** Characteristics of study participants aged 20–45 years (*n* = 118,924) ^a^.

Variables	All Subjects (*n* = 118,924)	Subjects without Anemia (*n* = 106,023)	Subjects with Anemia (*n* = 12,901)	*p*
Demographic and lifestyle data				
Age, years	33.6 ± 6.5	33.4 ± 6.5	35.3 ± 6.5	0.262
Gender				<0.001
Men	43,055 (36.2%)	42,543 (98.8%) ^b^	512 (1.2%) ^b^	
Women	75,869 (63.8%)	63,480 (83.7%) ^b^	12,389 (16.3%) ^b^	
Smoking				<0.001
Non-smoker	95,733 (80.5%)	83,842 (79.1%)	11,891 (92.2%)	
Past smoker	4617 (3.9%)	4352 (4.1%)	265 (2.1%)	
Current smoker	18,574 (15.6%)	17,829 (16.8%)	745 (5.7%)	
Drinking alcohol				<0.001
No	106,721 (89.7%)	94,461 (89.1%)	12,260 (95.0%)	
Yes	12,203 (10.3%)	11,562 (10.9%)	641 (5.0%)	
Chewing betel nut				<0.001
No	118,657 (99.8%)	105,761 (99.8%)	12,896 (99.9%)	
Yes	267 (0.2%)	262 (0.2%)	5 (0.1%)	
Sleep duration				<0.001
<6 h	98,970 (83.2%)	88,358 (83.3%)	10,612 (82.3%)	
6–8 h	19,554 (16.4%)	17,318 (16.3%)	2236 (17.3%)	
>8 h	400 (0.4%)	347 (0.4%)	53 (0.4%)	
Physical activity				<0.001
≤2 h/week	101,809 (85.6%)	90,347 (85.2%)	11,462 (88.8%)	
>2 h/week	17,115 (14.4%)	15,676 (14.8%)	1439 (11.2%)	
Prevalence of chronic diseases				
Hypertension	5446 (4.6%)	5058 (4.8%)	388 (3.0%)	<0.001
Diabetes	1538 (1.3%)	1378 (1.3%)	160 (1.2%)	0.258
Anthropometric measurements				
Body mass index, kg/m^2 c^				<0.001
Underweight	14,333 (12.1%)	12,414 (11.7%)	1919 (14.9%)	
Normal	72,838 (61.3%)	63,965 (60.3%)	8873 (68.8%)	
Overweight	20,348 (17.1%)	18,902 (17.8%)	1446 (11.2%)	
Obese	11,405 (9.5%)	10,742 (10.2%)	663 (5.1%)	
Central obesity ^d^	12,981 (10.9%)	12,048 (11.4%)	933 (0.9%)	<0.001
Anemia or inflammatory biomarkers				
Hemoglobin, mmol/L	8.6 ± 1.1	8.8 ± 0.8	6.8 ± 0.7	<0.001
Hematocrit, %	41.1 ± 4.6	41.9 ± 3.8	33.7 ± 2.9	<0.001
Red blood cells, 10^6^/μL	4.7 ± 0.5	4.7 ± 0.5	4.5 ± 0.6	<0.001
White blood cells, 10^3^/μL	6.0 ± 1.7	5.6 ± 2.2	6.1 ± 1.6	<0.001
C-reactive protein, nmol/L	18.8 ± 34.7	18.7 ± 40.5	18.9 ± 33.9	0.130

^a^ Continuous data are presented as mean ± SD, and categorical data are presented as number (percentage). The *p*-value was analyzed using a Mann–Whitney U test for continuous variables, and a chi-square test for categorical variables. ^b^ Percentages were calculated between those with and without anemia in the same gender. ^c^ Categories of body mass index, underweight: <18.5 kg/m^2^, normal: 18.5–23.9 kg/m^2^, overweight: 24.0–26.9 kg/m^2^, obese: ≥27 kg/m^2^. ^d^ Waist circumference ≥90 cm for men and ≥80 cm for women.

**Table 2:** Odds ratios and 95% confidence intervals for the associations between lifestyle, anthropometric variables, and anemia ^a^.

	Model 1		Model 2	
	OR (95% CI)	*p*	OR (95% CI)	*p*
Lifestyle				
Smoking (ref: non-smoker)				
Past smoker	0.29 (0.27, 0.32)	0.001	0.68 (0.63, 0.74)	0.001
Current smoker	0.43 (0.38, 0.49)	0.001	0.74 (0.64, 0.86)	0.001
Drinking alcohol (ref: no drinking)	2.55 (2.31, 2.81)	0.001	1.46 (1.32, 1.61)	0.001
Sleep duration (ref: 6–8 h)				
Short sleep duration (<6 h)	1.22 (0.91, 1.62)	0.188	1.12 (0.83, 1.50)	0.695
Long sleep duration (>8 h)	1.04 (0.98, 1.19)	0.140	1.01 (0.96, 1.07)	0.468
Inactive physical activity ^b^ (ref: >2 h/week)	1.38 (1.31, 1.46)	0.001	0.97 (0.89, 1.05)	0.433
Anthropometric measurements				
Body mass index ^c^ (ref: normal)				
Underweight	2.58 (2.33, 2.88)	0.001	1.20 (1.10, 1.42)	0.001
Overweight	1.24 (1.11, 1.38)	0.001	1.23 (1.10, 1.38)	0.001
Obese	2.27 (2.07, 2.49)	0.001	1.34 (1.22, 1.48)	0.001
Central obesity ^d^ (ref: normal)	1.64 (1.51, 1.77)	0.001	1.28 (1.18, 1.39)	0.001

^a^ Model 1 was unadjusted. Model 2 was adjusted for age, gender, hypertension, and diabetes. ^b^ Physical activity ≤2 h/week. ^c^ Categories of body mass index, underweight: <18.5 kg/m^2^, normal: 18.5–23.9 kg/m^2^, overweight: 24.0–26.9 kg/m^2^, obese: ≥27 kg/m^2^. ^d^ Waist circumference ≥90 cm for men and ≥80 cm for women.

**Table 3:** Multivariable regression models for associations between lifestyle, anthropometric variables, and anemia–inflammation-related biomarkers ^a^.

	Hb (mmol/L) β (95% CI)	Hct (%) β (95% CI)	RBC (10^6^/μL) β (95% CI)	WBC (10^3^/μL) β (95% CI)	CRP (nmol/L) β (95% CI)
Lifestyle					
Smoking (ref: non-smoker)					
Past smoker	0.18 (0.17, 0.19) *	0.09 (−0.10, 0.20)	−0.00 (−0.02, 0.01)	0.13 (0.07, 0.19) **	−0.29 (−1.51, 0.91)
Current smoker	0.29 (0.28, 0.32) **	0.74 (0.68, 0.80) **	0.00 (−0.01, 0.01)	0.68 (0.65, 0.71) **	1.21 (0.52, 1.89) **
Drinking alcohol (ref: no drinking)	−0.08 (−0.09, −0.06) **	0.06 (−0.01, 0.13)	−0.05 (−0.05, −0.04) **	−0.02 (−0.06, 0.02)	0.35 (−0.42, 1.13)
Sleep duration (ref: 6–8 h)					
Short sleep duration (<6 h)	−0.02 (−0.03, −0.01) *	−0.03 (−0.08, 0.02)	0.00 (−0.03, 0.04)	0.28 (0.03, 0.53) *	2.31 (−3.15, 7.77)
Long sleep duration (>8 h)	−0.03 (−0.10, 0.04)	−0.03 (−0.33, 0.27)	0.00 (−0.00, 0.01)	0.02 (−0.05, 0.01)	−0.24 (−0.92, 0.43)
Inactive physical activity ^b^ (ref: >2 h/week)	−0.01 (−0.02, −0.01) **	−0.33 (−0.39, −0.26) **	−0.03 (−0.03, −0.02) **	0.14 (0.11, 0.17) *	1.17 (0.55, 1.79) *
Anthropometric measurements					
Body mass index ^c^ (ref: normal)					
Underweight	−0.02 (−0.03, 0.01)	−0.22 (−0.91, 0.72)	−0.22 (−0.20, −0.23) **	−0.25 (−0.28, −0.22) **	0.77 (−0.14, 1.59)
Overweight	−0.05 (−0.07, −0.03) **	−0.45 (−0.53, −0.37) **	−0.02 (−0.03, −0.01) **	0.42 (0.38, 0.45) **	3.52 (2.86, 4.18) **
Obese	−0.13 (−0.15, −0.11) **	−0.86 (−0.93, −0.78) **	−0.09 (−0.10, −0.08) **	0.73 (0.68, 0.78) **	9.58 (8.43, 10.70) **
Central obesity ^d^ (ref: normal)	−0.14 (−0.15, −0.12) **	−0.78 (−0.84, −0.71) **	−0.06 (−0.07, −0.05) **	0.35 (0.31, 0.39) **	9.60 (8.67, 10.53) **

Hb: hemoglobin, Hct: hematocrit, RBC: red blood cells, WBC: white blood cells, CRP: C-reactive protein. ^a^ Model was adjusted for age, gender, hypertension, and diabetes. ^b^ Physical activity ≤2 h/week. ^c^ Categories of body mass index, underweight: <18.5 kg/m^2^, normal: 18.5–23.9 kg/m^2^, overweight: 24.0–26.9 kg/m^2^, obese: ≥27 kg/m^2^. ^d^ Waist circumference ≥ 90 cm for men and ≥80 cm for women. * *p* < 0.05. ** *p* < 0.001.

**Table 4:** Associations between tertiles of dietary pattern, anemia, and anemia-related biomarkers among adults in Taiwan ^a^.

	Anemia OR (95% CI)	Anemia-Related Biomarkers				
		Hb (mmol/L) β (95% CI)	Hct (%) β (95% CI)	RBC (10^6^/μL) β (95% CI)	WBC (10^3^/μL) β (95% CI)	CRP (nmol/L) β (95% CI)
Model 1 (ref: T1)						
T2	1.43 (1.36, 1.50) **	−0.26 (−0.28, −0.24) **	−1.19 (−1.26, −1.13) **	−0.12 (−0.13, −0.12) **	0.19 (0.17, 0.22) **	0.09 (0.09, 1.2) **
T3	1.87 (1.78, 1.95) **	−0.48 (−0.49, −0.46) **	−2.21 (−2.27, −2.15) **	−0.23 (−0.24, −0.23) **	0.34 (0.29, 0.41) **	1.90 (1.59, 2.60) **
Model 2 (ref: T1)						
T2	1.09 (1.01, 1.10) **	−0.11 (−0.12, −0.10) **	−0.12 (−0.13, −0.09) **	−0.00 (−0.01, −0.00) **	0.12 (0.00, 0.12) **	0.38 (−0.11, 1.03)
T3	1.10 (1.00, 1.23) **	−0.24 (−0.26, −0.24) **	−0.24 (−0.24, −0.09) **	−0.03 (−0.03, −0.02) **	0.17 (0.17, 0.21) **	1.70 (1.12, 2.37) **
Model 3 (ref: T1)						
T2	1.30 (1.27, 1.41) **	−0.01 (−0.02, −0.00) **	−0.01 (−0.10, −0.01) **	−0.01 (−0.01, −0.00) **	0.04 (0.01, 0.07) **	0.64 (0.19, 0.89) **
T3	1.59 (1.51, 1.67) **	−0.03 (−0.04, −0.02) **	−0.04 (−0.01, −0.09) **	−0.02 (−0.03, −0.02) **	0.08 (0.05, 0.10) **	1.64 (0.50, 2.21) **

Hb: hemoglobin, Hct: hematocrit, RBC: red blood cells, WBC: white blood cells, CRP: C-reactive protein. ^a^ Model 1 was unadjusted. Model 2 was adjusted for age and gender. Model 3 was adjusted for age, gender, smoking, drinking alcohol, sleep duration, physical activity, hypertension, diabetes, body mass index, and central obesity. ** *p* < 0.001.

## Data Availability

The data that support the findings of this study are available from the Mei Jau Health Management Institution, but restricted for research use only. The data are not publicly available.
